# Pneumococcal Pneumonia Vaccination Among Older African American and Hispanic Men With Chronic Conditions

**DOI:** 10.1093/geroni/igaa057.1081

**Published:** 2020-12-16

**Authors:** Matthew Smith, Ledric Sherman, Daunte Cauley, Brittany Badillo, R Kirby Goidel

**Affiliations:** Texas A&M University, College Station, Texas, United States

## Abstract

The CDC estimates that 70% of adults ages 65 years and older received the pneumococcal pneumonia vaccination (PPV). However, documented PPV rates are substantially lower for men, especially non-white men. This study examined common and unique factors associated with PPV among racial/ethnic minority men age 65 and older with one or more chronic conditions. Data were analyzed from a national sample of 470 African American (n=267) and Hispanic (n=203) males using an internet-delivered questionnaire. Two binary logistic regression models were fitted to compare factors associated with PPV. On average, participants were age 70.1(±4.5) years and reported 3.9(±2.6) chronic conditions. PPV rates were 56.8% and 43.2% among African American and Hispanic males, respectively. Across models, men who received vaccines for influenza (P<0.001) and shingles (P<0.01) were more likely to receive the PPV; whereas, those who reported more disease self-care barriers were less likely to receive the PPV (P<0.05). Among African American males, those who were widowed (OR=3.80, P=0.022) and had an annual eye examination (OR=3.10, P=0.001) were more likely to receive the PPV; whereas, divorced/separated men were less likely to receive the PPV (OR=0.33, P=0.003). Among Hispanic males, those who took more medications daily (OR=1.36, P=0.005), reported higher disease self-management efficacy (OR=1.15, P=0.011), and had a colon cancer test in the past 12 months (OR=3.55, P=0.007) were more likely to receive the PPV. Findings suggest the need for culturally tailored education and self-management interventions to increase PPV and preventive healthcare service utilization among older racial/ethnic minority men.

